# Benign Vulvar Adnexal Tumours - A 5 year Study in a Tertiary Care Hospital

**Published:** 2015-11

**Authors:** Kanwardeep Kaur Tiwana, Sarita Nibhoria, Harpreet Kaur, Akanksha Bajaj, Richa Phutela

**Affiliations:** 1 Department of Pathology, Guru Gobind Singh Medical College, Faridkot, Punjab, India; 2 Department of Obstetrics and Gynaecology, Guru Gobind Singh Medical College, Faridkot, Punjab, India

**Keywords:** Vulvar Adnexal Tumours, Hidradenoma Papilliferum, Syringoma

## Abstract

Despite of the fact that the vulva contains a high density of apocrine and anogenital mammary glands in addition to eccrine glands and folliculosebaceous units, the benign vulvar adnexal tumours are rare. Though the varied clinical presentation and diverse histopathological spectrum of vulvar neoplasms has amazed the pathologists, only few studies have been reported in literature. The present five year study consists of only five cases of benign vulvar neoplasms depicting their rarity. Hidradenoma papilliferum and syringoma were the most common entities followed by Chondroid syringoma. The aim of our study is to explore and highlight the histopathological diversity of benign vulvar adnexal tumours reflecting the relative frequency of these structures.

## Introduction

The vulva is composed of labia majora, labia minora, clitoris, vulvar vestibule, vestibulovaginal bulbs, urethral meatus, Bartholin's and Skene's glands. Also vulvar region contains high density of apocrine and anogenital mammary like glands in addition to eccrine glands and pilosebaceous units. However despite the high density of these units the tumours derived from them are very low. The incidence of benign vulvar adnexal tumours has not been highlighted much. The aim of the present study is to highlight these benign vulvar adnexal tumours so that it would reflect the relative frequency of these structures (1).

## Materials and methods

In the present five year study all the cases presenting with vulvar lesions were consecutively screened during the period of 2010 to 2015. The haematoxylin and eosin stained histopathological sections of vulvar biopsies in the Pathology department were reevaluated. Physical and vulvar examination details were obtained from the patient files and histopathology requisition form. The clinical presentation and histopathological details were evaluated and diagnosis was confirmed. As the diagnosis was made purely on histopathological grounds, no ancillary aids were required

## Results

In the present five year study a total of five cases of benign vulvar adnexal tumours were identified. The patient age ranged between 22-60 years with mean age of 42.8 years. Hidradenoma papilliferum (40%) and Syringoma (40%) were the most common tumours followed by Chondroid syringoma (20%) on histopathological examination. 

**Table 1:** Characteristics of reported cases

Age	Clinical presentation	Histopathological Diagnosis	Percentage
**42 years**	**Indurated cyst**	**Hidradenoma papilliferum**	**40%**
**32 years**	**Sessile polyp**	**Hidradenoma papilliferum**	
**60 years**	**Pruritus**	**Syringoma**	**40%**
**40 years**	**Pruritus**	**Syringoma**	
**22 years**	**Painful tender lesion**	**Chondroid syringoma**	**20%**

A wide range of clinical presentation was seen in all the five cases and important point was that tumour was not clinically suspected. Syringomas (40%) presented with pruritus, Hidradenoma papilliferum presented as indurated cyst and sessile polyp and Chondroid syringoma presented as painful nodular lesion (Table 1).

***Hidradenoma papilliferum***

**Figure 1:**
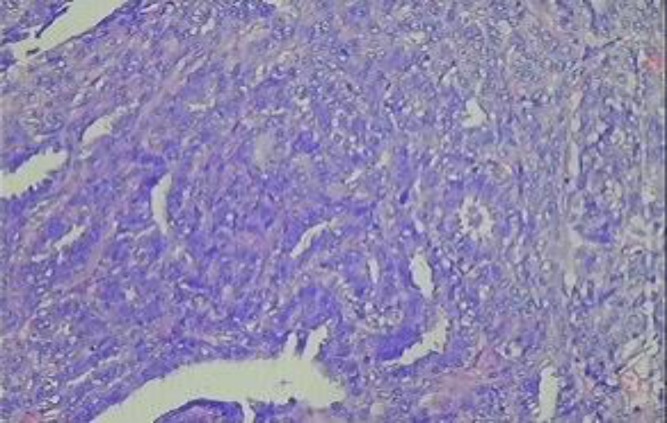
Hidradenoma Papilliferum (H&E X100)

It is derived from anogenital mammary glands. Histopathologically the tumour has papillary and glandular architecture. The epithelial cells are columnar having pale eosinophilic cytoplasm and are surrounded by myoepithelial layer (Figure 1).

***Syringoma***

**Figure 2:**
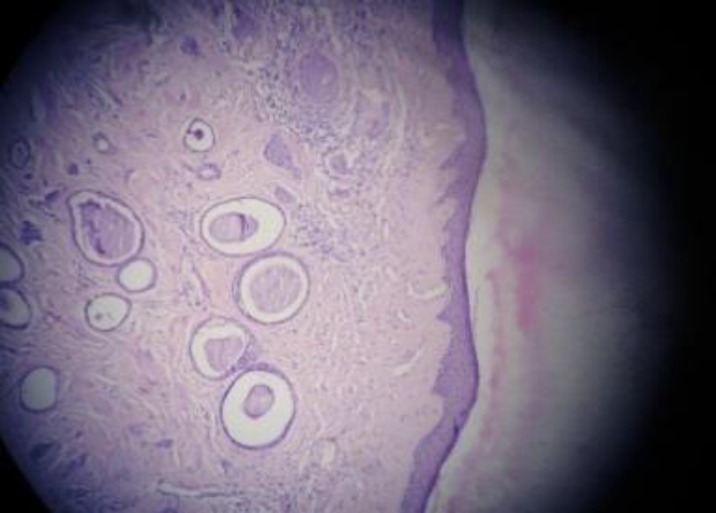
Syringoma (H&E X100)

It is a benign eccrine neoplasm. Histopathologically it consists of small epithelial cysts and dilated duct like spaces lined by two rows of cells, inner epithelial and outer myoepithelial cells. The ductular structures form comma like structures (Figure 2).

***Chondroid Syringoma (Mixed tumour of vulva)***

**Figure 3:**
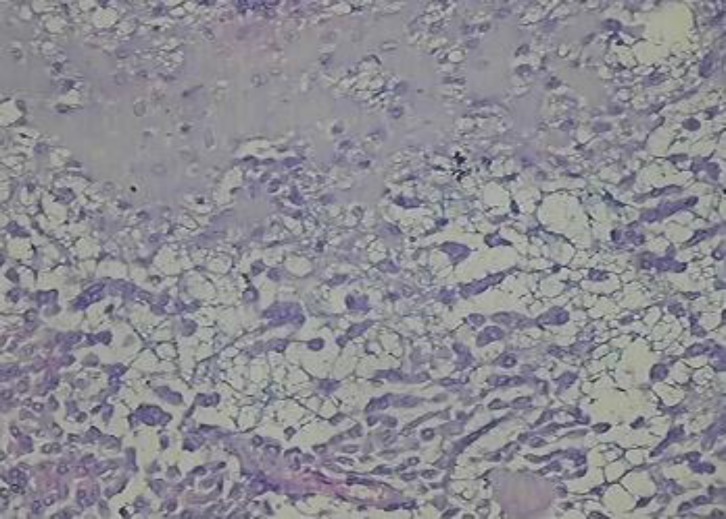
Chondroid Syringoma exhibiting cartilaginous differentiation (H&E X450)

It is a rare tumour of vulva arising from sweat glands or Bartholin's glands. Histopathologically the tumour contains structures of epithelial and myoepithelial cells associated with myxoid or cartilaginous structures (Figure 3).

## Discussion

Vulvar region contains dense apocrine glands, anogenital mammary like glands along with eccrine glands and folliculosebaceous units. However the frequency of adnexal tumours (benign as well as malignant) is very low. Their incidence has not been reported much. This field despite being uncommon has not been explored. The aim of this study is to highlight benign adnexal tumours of the vulva as the spectrum of various vulvar adnexal lesions appears to reflect the frequency of the underlying glandular elements.

There have been very few studies done which have addressed this issue. Only one study done by Baker et al objectively evaluated the vulvar adnexal lesions, they retrospectively retrieved a 32 year data and reevaluated all the vulvar adnexal lesions consisting of 189 cases. 2 In this study we evaluated only the benign adnexal vulvar tumours over the period of 5 years and total of five cases were found. The patient age ranged between 22-60 years with mean age being 42.8 years.

Hidradenoma papilliferum and syringoma were the most common tumours which is in discordance with study done by Baker et al where Hidradenoma papilliferum (60%) was the most common tumour followed by syringoma (22%) (2).

Hidradenoma papilliferum was first reported by Worth in 1878 and the most common sites are vulva and perianal regions (3). This tumour is derived from anogenital mammary like glands (4-7). In vulva the sites of involvement include labia minora (50%), labia majora (40%), fourchette (7%) and clitoris (3%) (8). This distribution directly reflects the distribution of anogenital mammary like glands.

Syringoma is a benign eccrine gland tumour and it is very rare. But in the present study it was the most common tumour along with Hidradenoma papilliferum. It presented as pruritic lesion in all cases which is the most common presentation in the study done by Huang et al (9). In the present study all cases were incidental findings. This is in concordance with study done by Baker et al where 41% of cases of syringoma were incidental findings. These observations suggest that prevalence of syringomas may be underestimated as they were mostly asymptomatic.

Chondroid syringoma is a benign mixed tumour and has histological features of mixed tumour of salivary gland. It is a very rare tumour with only few cases reported in literature (10- 14). These tumours are considered benign but due to paucity of data it is difficult to determine the natural history at this site.

## Conclusion

The spectrum of various vulvar tumours reflects the relative frequency of the underlying glandular elements. This study is done to explore the field which is rare and has not been explored much so that natural progression of these tumours could be assessed.

## References

[1] Williams PL, Bannister LH, Berry MM (1995). Gray’s Anatomy.

[2] Baker GM, Selim MA, Hoang MP (2013). Vulvar Adnexal Lesions: A 32-Year, Single- Institution Review From Massachusetts General Hospital. Arch Pathol Lab Med.

[3] Woodworth H, Docketty MB, Wilson RB, Pratt JH (1971). Papillary Hidradenoma of the vulva: a clinicopathologic study of 69 cases. Am J Obstet Gynecol.

[4] Parks A, Branch KD, Metcalf J, Underwood P, Young J (2012). Hidradenoma papilliferum with mixed histopathologic features of syringocystadenoma papillerum and anogenital mammary –like glands: report of a case and review of the literature. AM J Dermatopathol.

[5] Nishie W, Sawamura D, Mayuzumi M, Takahashi S, Shimizer H (2004). Hidradenoma papilliferum with mixed histopathologic features of syringocystadenoma papilliferum and anogenital mammary-like glands. J Cutan Pathol.

[6] vander Putte SC (1994). Mammary like glands of the vulva and their disorder. Int J Gynecol Pathol.

[7] vander Putte SC, van Gorp LH (1994). Adenocarcinoma of the mammary- like glands of the vulva: a concept unifying sweat gland carcinoma of the vulva, carcinoma of supernumerary mammary glands and extramammary Paget’s disease. J Cutan Pathol.

[8] Scurry J, vander Putte SC, Pyman J, Chetty N, Szabo R (2009). Mammary like gland adenoma of the vulva: a review of 46 cases. Pathology.

[9] Huang YH, Chuang YH, Kuo TT, Yang LC, Hong HS (2003). Vulvar syringoma: a clinicopathologic and immunohistochemical study of 18 patients and results of treatment. J Am Acad Dermatol.

[10] Ordonez NG, Manning JT, Luna MA (1981). Mixed tumour of the vulva: a report of two cases probably arising in Bartholin’s gland. Cancer.

[11] Dykgraaf RH, van Veen MM, van Bekkum-de Jonge EE, Gerretsen J, de Jong D, Burger CW (2006). Pleomorphic adenoma of the vulva: a review illustrated by a clinical case. Int J Gynecol Cancer.

[12] Rorat E, Wallach RC (1984). Mixed tumors of the vulva: clinical outcome and pathology. Int J Gynecol Pathol.

[13] Wilson D, Woodger BA (1974). Pleomorphic adenoma of the vulva. J Obstet Gynaecol Br Commonw.

[14] Chome J, Giard R (1956). Case report of an unusual tumor of the vulva: epithelioma of rearranged stroma or so- called mixed tumor [in French]. Bull Fed Soc Gynecol Obstet Lang Fr.

